# Potential Diagnostic and Prognostic Value of Neutrophil Counts, Neutrophil–Lymphocyte Ratio, Pan-Immunoinflammatory Score, Systemic Immunoinflammatory Index, and Systemic Inflammatory Response Index in Brain Tumors

**DOI:** 10.3390/diagnostics16060933

**Published:** 2026-03-21

**Authors:** Murat Ozcan Yay, Adem Keskin, Mahmut Ali Osman Eryilmaz, Cagatay Kaya

**Affiliations:** 1 Department of Neurosurgery, Faculty of Medicine, Aydın Adnan Menderes University, Aydin 09100, Turkey; cagatay646@gmail.com; 2 Department of Medical Biochemistry, Faculty of Medicine, Aydin Adnan Menderes University, Aydin 09100, Turkey; adem.keskin@adu.edu.tr; 3 Department of Neurology, Faculty of Medicine, Aydın Adnan Menderes University, Aydin 09100, Turkey; mahmut.eryilmaz@adu.edu.tr

**Keywords:** brain tumor, neutrophil, neutrophil–lymphocyte ratio (NLR), pan-immunoinflammatory value (PIV), systemic immunoinflammatory index (SII), systemic inflammatory response index (SIRI), neurological deficit, karnofsky performance status (KPS), recurrence, motor weakness

## Abstract

**Background/Objectives**: The aim of this study is to investigate the possible association between systemic inflammation parameters and tumor presence in brain tumor patients with high morbidity and mortality rates, to examine the discriminative performance of these markers and, furthermore, to investigate the relationship of these markers with the clinical findings of patients at different time points. **Methods**: This study included 99 patients with brain tumors as the case group and 99 healthy individuals as the control group. Neutrophil, lymphocyte, monocyte, and platelet levels, as well as indices and ratios derived from these parameters, were compared among the participants. Binary logistic regression and ROC analyses were applied to variables showing significant differences, and the relationship between these variables and demographic and clinical findings was also evaluated. **Results**: The neutrophil count, neutrophil–lymphocyte ratio (NLR), pan-immunoinflammatory value (PIV), systemic immunoinflammatory index (SII), and systemic inflammatory response index (SIRI) values of the case group were higher compared to the control group. While these parameters were associated with the presence of brain tumors, the highest odds ratio, area under the curve, and specificity were found in the neutrophil count, and the highest sensitivity was found in the SIRI parameter. Some or all of these parameters differed according to tumor type, localization, motor weakness, 3-month adjuvant treatment, 6-month recurrence, postoperative day 1, 3-month and 6-month neurological deficits and 3-month and 6-month Karnofsky Performance Status. **Conclusions**: These parameters can be considered useful biomarkers that show moderate discrimination in patients with brain tumors and can support clinical evaluation.

## 1. Introduction

Brain tumors, including primary gliomas and brain metastases, are among the deadliest tumors because effective macromolecular antitumor drugs cannot easily cross the blood–brain tumor barrier and the blood–brain barrier [1]. Despite their relative rarity, these tumors lead to disproportionately high morbidity and mortality and possess numerous histologically distinct subtypes [2]. Although brain tumors have many different histological subtypes, they can generally impair the brain’s ability to regulate its functions. These functions include motor functions, consciousness, sensory perception, cognitive processes, speech, and memory. This impairment occurs due to the tumor’s location and its tendency to invade surrounding brain tissue. Neurological symptoms such as epileptic seizures, headaches, and sensory loss are frequently seen in relation to the tumor’s location and infiltrative growth pattern. This situation leads to varying degrees of cognitive and functional impairment in patients [3].

Inflammation and the innate immune system play a central role in cancer development, including those affecting the central nervous system. Treatment approaches for most brain and spinal cord tumors rely on surgical resection, radiotherapy, and chemotherapeutic agents; however, despite decades of advancement, the clinical outcomes of these treatments remain inadequate. Recent studies have revealed a strong link between inflammation and tumor formation, highlighting the complex nature of bidirectional interactions between immune and tumor cells and the anti-tumor and pro-tumor effects of inflammation [4].

In central nervous system tumors, increasing evidence regarding the modulation of the immune response by cancer cells suggests that the reciprocal communication between immune cells and tumor cells supports tumor survival and growth by creating an immune-suppressed tumor microenvironment [4]. In this process, while the immune system exhibits anti-tumor effects in the early stages, as the disease progresses, tumors can utilize the normally therapeutic mechanisms of the inflammatory response to their advantage. The infiltration of various immune cells into the developing tumor tissue contributes to the formation of a pro-inflammatory tumor microenvironment [5]. In this context, assessing the systemic immune-inflammatory status in a heterogeneous population of brain metastases is of great importance, particularly in terms of the bidirectional communication between the brain and the immune system in patients requiring neurosurgical resection [6].

It is hypothesized that there is a common biological mechanism underlying certain symptoms reported by cancer patients. Current multi-platform studies have shown that these symptoms may be triggered by proinflammatory toxicity resulting from the spread of local inflammation into the systemic circulation, which in turn may lead to significant changes in immune, metabolic, and nervous system functions and activities [7]. This suggests that systemic inflammation in cancer may play a decisive role not only in the development of symptoms but also in the course and prognosis of the disease. In this context, markers reflecting systemic inflammation, such as the neutrophil–lymphocyte ratio (NLR), pan-immunoinflammatory value (PIV), systemic immunoinflammatory index (SII), and systemic inflammatory response index (SIRI) have recently been investigated as factors associated with prognosis in many tumors, including head and neck, bladder, breast, prostate, stomach, and lung cancers [8,9,10,11,12,13]. However, most existing studies have evaluated these markers in isolation, and there are very few studies that have comparatively examined multiple inflammation indices within the same patient cohort. Evaluating these indices together within the same patient cohort may help determine the relative discriminatory performance of different indices and reveal their complementary potential clinical roles. In particular, the combined evaluation of these multi-parameter indices, which reflect the systemic inflammatory response in central nervous system tumors, may contribute to a more comprehensive understanding of the inflammation–tumor interaction.

The aim of this study is to investigate the possible relationship between systemic inflammatory markers obtained from hematological parameters in patients diagnosed with brain tumors and the presence of brain tumors, as well as the discriminatory performance (sensitivity and specificity) of these markers. Additionally, it aims to evaluate the relationship between these markers and the patients’ demographic and tumor characteristics, as well as the clinical findings obtained during follow-up examinations on day 1, month 3, and month 6 in the early postoperative period.

## 2. Materials and Methods

### 2.1. Study Design

Ethical approval required for the study was obtained from the Non-Interventional Clinical Research Ethics Committee of Aydın Adnan Menderes University Faculty of Medicine (Protocol number: 2025/368, Decision no: 16, Approval date: 4 December 2025). After obtaining approval from the Ethics Committee, the required sample size for the study was calculated using G*Power version 3.1.9.7 (G*Power, Heinrich Heine University Düsseldorf, Düsseldorf, Germany). According to a power analysis, a minimum of 92 participants per group (total *n* = 184) was required with a one-way significance level of 0.05, an effect size of 0.5, and 95% power for the Wilcoxon–Mann–Whitney U test. Accordingly, the study’s case group consisted of 99 patients who presented to and underwent surgery for brain tumors at the Neurosurgery Outpatient Clinic of Aydın Adnan Menderes University Faculty of Medicine between 15 June 2023 and 15 June 2025. Preoperative evaluation included a neurological examination and radiological imaging (primarily magnetic resonance imaging) and suggested the presence of a brain tumor. Based on the neurosurgical evaluation, surgical resection or biopsy was performed. Tumor diagnoses were confirmed by histopathological examination of surgical specimens obtained during tumor resection or biopsy. Tumors were classified according to the 2021 World Health Organization (WHO) Classification of Tumors of the Central Nervous System, which combines histological and molecular characteristics [14]. Diffuse gliomas were categorized as low-grade or high-grade according to WHO grading criteria. The glioblastoma cases included in the study were diagnosed according to the 2021 WHO criteria and were consistent with isocitrate dehydrogenase (IDH) wild-type glioblastoma. Molecular parameters, such as IDH mutation status, were considered when available as part of routine pathological evaluation. Patients with metastatic tumors included in the study had only one localization; patients with multiple metastases were excluded. Additionally, exclusion criteria for the study included individuals with a history of acute infection, autoimmune disease, chronic inflammatory condition, or those who had received corticosteroid treatment. In addition, a control group of 99 healthy individuals with similar average age and gender ratios to the case group was included. The descriptive characteristics of the patients in the case group and their clinical data before surgery and at 3 and 6 months postoperatively are presented in Table 1.

### 2.2. Study’s Data

Before participants’ data were collected, participants and/or their relatives were informed about the purpose and scope of the study, and written informed consent was obtained. After obtaining consent, patient data were retrospectively retrieved from the Hospital Information Management System (HIMS). Complete blood count data for the case group were obtained from blood samples taken from all patients preoperatively and at hospital admission to ensure homogeneity at the time of sampling. NLR, monocyte/lymphocyte ratio (MLR), and platelet/lymphocyte ratio (PLR) were calculated from complete blood count results. In addition, PIV, SII, and SIRI were calculated as previously described [15,16,17,18] using the following formulas:

PIV = (Neutrophil × Platelet × Monocyte)/Lymphocyte SII = (Neutrophil × Platelet)/Lymphocyte SIRI = (Neutrophil × Monocyte)/Lymphocyte.

All hematological parameters were calculated using absolute cell counts obtained from complete blood count analyses.

### 2.3. Statistical Analysis

Statistical analysis of the data obtained from the participants was performed using SPSS version 22.0 for Windows (IBM Corp., Armonk, NY, USA). Continuous variables were expressed as mean ± standard deviation ($\bar{X}$ ± SD), and categorical variables were expressed as number and percentage (*n* (%)). Normality of continuous variables was assessed using the Shapiro–Wilk test, and homogeneity of variance was assessed using the Levene test. Mann–Whitney U test and Kruskal–Wallis test were used to compare continuous variables. Correlation analyses were performed using the Spearman correlation test. The chi-square test was used to compare categorical variables. Bonferroni correction was applied in multiple comparisons. A *p*-value < 0.05 was considered statistically significant.

## 3. Results

The study included 99 patients aged 18–86 years who presented to the Neurosurgery Outpatient Clinic of Aydın Adnan Menderes University Hospital with a diagnosis of brain tumor as the case group. The control group consisted of 99 individuals in the same age range who did not have any chronic health problems. The mean age of the case group was 60.33 ± 15.10, while in the control group it was 60.30 ± 12.42. Furthermore, the percentage of male individuals in the case group was 54.55% (*n* = 54), and the percentage of female individuals was 45.45% (*n* = 45), while in the control group these percentages were 61.62% (*n* = 61) and 38.38% (*n* = 38), respectively. There was no significant difference between the two groups in terms of mean age and gender ratios (*p* = 0.988 and *p* = 0.313, respectively). Laboratory findings for these groups are presented in Table 2.

In the case group, the mean values of neutrophil count and NLR, PIV, SII, and SIRI were found to be higher compared to the mean values of the control group. Of the 99 patients in the case group, 25 were diagnosed with metastatic brain tumors and 74 with primary brain tumors (Table 1). No statistically significant difference was observed between patients with primary and metastatic brain tumors in terms of mean neutrophil count, NLR, PIV, SII, and SIRI values (*p* = 0.114, *p* = 0.071, *p* = 0.430, *p* = 0.058, *p* = 0.459, respectively). Because no statistically significant difference was observed between patients with primary and metastatic brain tumors, they were analyzed as a single cohort in the subsequent analyses. Binary logistic regression analysis was performed to evaluate the relationship between these parameters and the presence of brain tumors. Additionally, ROC analysis was applied to determine the discriminative performance (sensitivity and specificity) of these parameters. The results of these two analyses are presented in Table 3.

**Figure 1:**
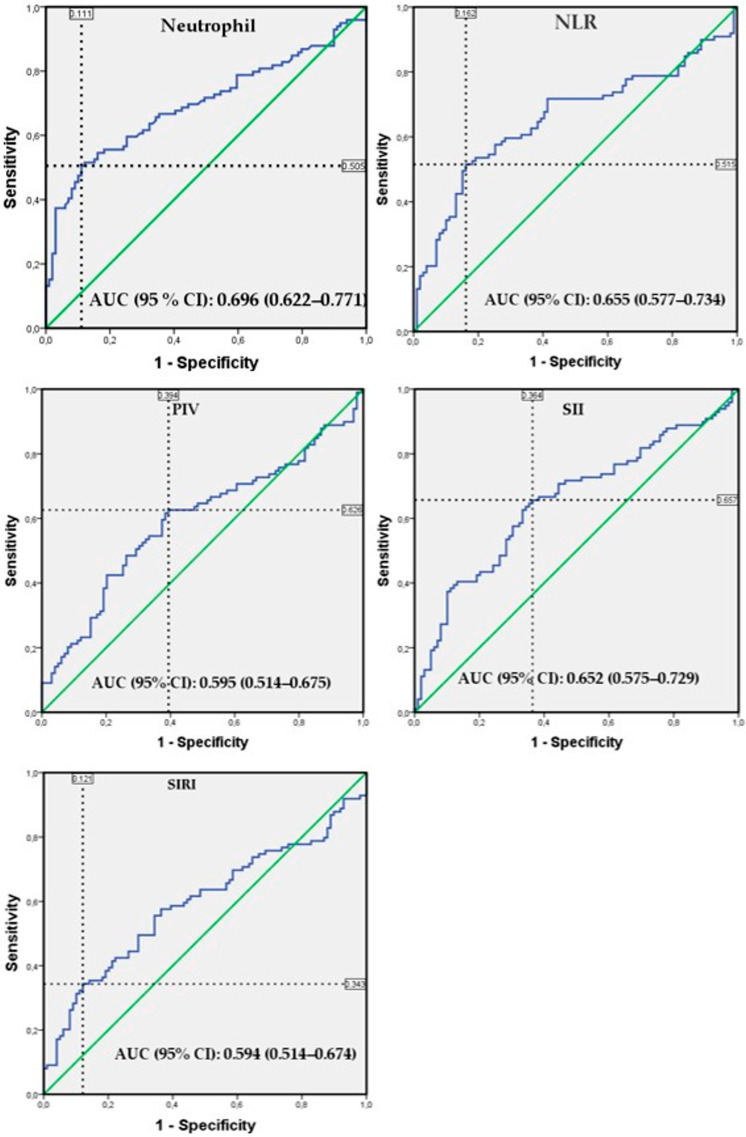
Receiver operating characteristic (ROC) curves of parameters associated with brain tumor presence. The blue line shows the ROC curve for each parameter, the green diagonal line shows the reference line (AUC = 0.5), and the black dotted lines show the optimal cutoff point corresponding to sensitivity and 1-specificity.

The analyses revealed that all five parameters examined showed a statistically significant association with the presence of brain tumors (Table 3). According to the binary logistic regression analysis, the highest odds ratio was obtained with neutrophil count, while according to the ROC analysis results, the highest area under curve (AUC) and specificity were again found in neutrophil count, and the highest sensitivity was found in the SIRI parameter (Table 3 and Figure 1). In addition, the fact that the AUC confidence intervals of the five parameters largely overlap indicates that there is no statistically significant difference between the parameters (Table 3). On the other hand, pairwise comparison of ROC curves using the DeLong test revealed that the neutrophil count parameter had significantly higher AUC values than the PIV and SIRI parameters (*p* < 0.001). Additionally, the AUC values obtained from the five parameters are modest, and the sensitivity and specificity values can be considered moderate (Table 3).

Statistical analysis was performed to evaluate the relationship between statistically significant parameters found between the two groups and the demographic characteristics and clinical findings of the patients. Correlation analysis was performed to evaluate the relationship between these parameters and continuous or ordinal variables.

Neutrophil values were found to be negatively correlated with both the 3rd and 6th month Karnofsky Performance Status (KPS) scores (Table 4). NLR values showed a positive correlation with intraoperative blood loss and a negative correlation with the 3rd month KPS score (Table 4). The PIV showed a negative correlation with both the 3rd and 6th month KPS scores (Table 4). The SII value showed a positive correlation with intraoperative blood loss (Table 4). The SIRI value showed a positive correlation with tumor size (anterior–posterior) and a negative correlation with both the 3rd and 6th month KPS scores and surgery duration (Table 4).

Statistically significant differences were found in neutrophil count, NLR, PIV, SII, and SIRI values among subgroups formed according to tumor types (Table 5). Statistically significant differences were found in neutrophil count, NLR, PIV, SII, and SIRI values among subgroups formed according to tumor localization (Table 5). Statistically significant differences were found in neutrophil count, NLR, PIV, SII, and SIRI values among subgroups formed according to adjuvant therapy (3 months) (Table 5). On the other hand, no statistically significant differences were found in neutrophil count, NLR, PIV, SII, and SIRI values among subgroups formed according to adjuvant therapy (6 months) (Table 5).

In patients with motor weakness, neutrophil count, NLR, PIV, and SII values were found to be statistically significantly higher compared to patients without motor weakness (Table 5). On the other hand, there was no significant difference in these values between patients with speech impairment and those who were not (Table 5).

In patients who developed new neurological deficit (Postoperative day 1), neutrophil count, NLR, and SII values were found to be statistically significantly higher than in patients without these deficits (Table 5). Similarly, at 3 and 6 months, neutrophil count, NLR, PIV, and SII values were found to be statistically significantly higher in patients with neurological deficit compared to patients without neurological deficit (Table 5).

At 3 months, there were no significant differences in neutrophil count, NLR, PIV, SII, or SIRI values among patients according to recurrence status; however, at 6 months, neutrophil counts were significantly higher in patients with recurrence compared to those without recurrence (Table 5). No significant differences were found between patients based on re-surgery status in neutrophil count, NLR, PIV, SII, or SIRI values (Table 5).

## 4. Discussion

Neutrophils, one of the most abundant immune cells in circulation, frequently infiltrate tumor tissue at high rates. Neutrophils associated with brain tumors, which exhibit immunosuppressive and pro-angiogenic properties, have a distinct inflammatory signature driven by tumor necrosis factor alpha, ceruloplasmin, and other soluble inflammatory mediators. This supports the existence of a critical myeloid niche involved in regulating general immune suppression in human brain tumors [19]. Despite their functional heterogeneity, the high density of intratumoral neutrophils is associated with poor clinical outcomes in central nervous system tumors, as in many other cancer types. The tumor microenvironment promotes tumor growth, invasion, and the development of treatment resistance by reprogramming neutrophils. In this context, neutrophils emerge as an important component of the immunosuppressive tumor microenvironment, and this situation is considered one of the main mechanisms limiting the clinical efficacy of promising immunotherapeutic approaches [20]. This reprogramming process, particularly through metabolic and epigenetic mechanisms such as increased glucose metabolism and histone lactation in hypoxic regions, leads to increased expression of immunosuppressive genes and suppression of antitumor immune responses [21]. Hypoxia-dependent tumor niches have been shown to be strongly associated with poor prognosis. Furthermore, the Tumor Structure Score reveals that highly organized tumors, characterized by well-defined vascular structure and prominent hypoxic niches, are associated with worse survival outcomes [22].

This study found higher neutrophil counts in patients with brain tumors compared to healthy individuals. Statistical analyses also revealed a 1.329 odds ratio, a 0.696 AUC value, and an 88.89% specificity rate between tumor presence and neutrophil count. Furthermore, neutrophil counts showed a negative correlation with both 3rd and 6th month KPS scores, and differences were observed depending on tumor type, tumor localization, and adjuvant therapy (3 months). In addition, neutrophil counts were found to be higher in patients who experienced motor weakness on the first day of post-surgery and developed new neurological deficits compared to those who did not experience these conditions. Similarly, at 3 and 6 months, neutrophil counts were higher in patients with neurological deficits compared to those without. Finally, neutrophil counts were higher in patients with recurrence (6 months) compared to those without recurrence. On the other hand, considering both the AUC value and effect size, neutrophil counts show a moderate predictive performance.

In a recent study, it was reported that approximately three-quarters of patients with low-grade brain tumors treated with radiotherapy alone had increased NLR despite radiotherapy [23]. Another recent study examining the role of neutrophils in glioma and brain metastases suggests that NLR may be a potential biomarker for brain cancer. While the underlying biological mechanisms of the relationship between high NLR and poor prognosis in cancer patients are not yet fully elucidated, high NLR in peripheral blood has recently been reported as a negative prognostic indicator. However, it has also been reported that NLR may be helpful in assessing the early effects of systemic treatment. On the other hand, since acute conditions such as bacterial or viral infections and drug therapies can overlap with chronic inflammatory processes and affect neutrophil and lymphocyte counts, the clinical use and interpretation of NLR requires a careful validation process [24]. Furthermore, a study by Ashwath et al. reported that NLR is related to the histopathological grade of brain tumors, and significant changes in NLR values were observed as the tumor grade increased [25].

This study found higher NLR in patients with brain tumors compared to healthy individuals. Statistical analyses also revealed a 1.195 odds ratio, a 0.655 AUC value, and an 83.84% specificity rate between tumor presence and NLR. Furthermore, NLR showed a negative correlation with 3rd month KPS scores and a positive correlation with intraoperative blood loss, and differences were observed depending on tumor type, tumor localization, and adjuvant therapy (3 months). In addition, NLR were found to be higher in patients who experienced motor weakness on the first day of post-surgery and developed new neurological deficits compared to those who did not experience these conditions. Similarly, at 3 and 6 months, NLR were higher in patients with neurological deficits compared to those without. On the other hand, the AUC value obtained from NLR values, like neutrophil counts, also shows a moderate level of discriminatory capacity. On the other hand, considering both the AUC value and effect size, NLR values such as neutrophil count also show a moderate level of predictive performance.

Recent studies have examined PIV in various inflammatory and cardiovascular conditions and have shown that these markers have broader systemic effects [26,27]. Similarly, a recent study evaluating a cohort of 89 patients with high-grade glioma reported that PIV was able to effectively divide newly diagnosed high-grade glioma patients into two groups that differed significantly in terms of progression-free survival and overall survival [28]. Another recent study evaluating a cohort of 3856 patients diagnosed with brain metastases reported that the PIV is an independent prognostic factor for overall survival and that this marker should be included in the Graded Prognostic Assessment system [29].

This study found higher PIVs in patients with brain tumors compared to healthy individuals. The PIV showed a negative correlation with both the 3rd and 6th month KPS scores, and differences were observed depending on tumor type, tumor localization, and adjuvant therapy (3 months). In addition, PIVs were found to be higher in patients who experienced motor weakness compared to those who did not experience these conditions. At 3 and 6 months, PIVs were higher in patients with neurological deficits compared to those without. On the other hand, considering both the AUC value and effect size, PIVs, like neutrophil count and NLR values, also show a moderate predictive performance.

A recent meta-analysis encompassing twenty-one studies reported that SII and SIRI indices had no association with glioblastoma survival; however, it highlighted that NLR is a clinically useful and practical peripheral inflammatory marker for predicting glioblastoma prognosis [30]. On the other hand, another recent study analyzing 230 cases with brain metastasis reported that brain metastases cause disruption in the systemic immune-inflammatory balance and that these patients have higher SII indices compared to a healthy control [6]. In a recent study involving sixty-eight patients with high-grade glioma, the AUC value for SII in predicting early postoperative recurrence was reported as 0.781, sensitivity 75.0%, and specificity 72.9% [31].

This study found higher SII values in patients with brain tumors compared to healthy individuals. Statistical analyses also revealed a 1.052 odds ratio, a 0.652 AUC value, a 65.66% sensitivity rate, and a 63.64% specificity rate between tumor presence and SII values. Furthermore, SII values showed a positive correlation with intraoperative blood loss, and differences were observed depending on tumor type, tumor localization, and adjuvant therapy (3 months). Furthermore, in this study, the highest mean SII value was found in patients with adenocarcinoma metastasis, followed by those with neuroendocrine carcinoma metastasis. Furthermore, the mean SII value calculated in glioblastoma patients was below the overall mean for all brain tumor patients, while the mean SII value determined in high-grade diffuse glioma patients was above the overall mean. In addition, SII values were found to be higher in patients who experienced motor weakness on the first day post-surgery and developed new neurological deficits compared to those who did not experience these conditions. Similarly, at 3 and 6 months, SII values were higher in patients with neurological deficits compared to those without. On the other hand, considering both the AUC value and effect size, SII values, like neutrophil count, NLR and PIVs, also show a moderate predictive performance.

A recent meta-analysis encompassing 10 studies with 1942 participants reported that a high SIRI value is a significant predictor of overall survival and disease-free survival in patients with glioma and is a promising prognostic biomarker in glioma clinical practice [32]. A study examining 271 patients with brain metastases from lung cancer reported that SIRI was an independent prognostic factor for increased risk of death. They also established a cut-off value of 2.95 for this factor [33].

This study found higher SIRI values in patients with brain tumors compared to healthy individuals. Statistical analyses also revealed a 1.200 odds ratio, a 3.27 cut-off value, a 34.34% sensitivity rate (lowest among 5 parameters), and an 87.88% specificity rate (highest among 5 parameters) between tumor presence and SIRI values. Furthermore, SIRI values showed a positive correlation with tumor size (anterior–posterior) and a negative correlation with both the 3rd and 6th month KPS scores and surgery duration, and differences were observed depending on tumor type, tumor localization, and adjuvant therapy (3 months). On the other hand, considering both the AUC value and effect size, SIRI values, like others, show a moderate level of predictive performance.

Although the AUC values for these five parameters largely overlapped in this cohort, the DeLong test showed that the neutrophil count had a statistically significantly higher AUC value compared to the PIV and SIRI. This suggests that the neutrophil count, a simple parameter, may provide additional diagnostic value beyond that of more complex composite indices. However, the moderate AUC, sensitivity, and specificity values indicate that these parameters are not strong discriminatory factors on their own and that the aforementioned indices may be complementary in nature.

When the study’s effect size, AUC, sensitivity, and specificity values are evaluated together, it is observed that the five parameters examined demonstrate moderate predictive performance. This suggests that these markers may have potential value in terms of clinical discriminatory power. However, the current findings are considered more hypothesis-generating than definitive, and they require validation through larger, prospective studies.

Although the sample size was determined by performing a power analysis for the study, the heterogeneous nature of brain tumors can be considered a limitation of the study. Inclusion of multiple tumor subtypes in the case group leads to significant biological heterogeneity; furthermore, the limited sample size for subgroup analyses restricts a detailed assessment of potential differences specific to tumor subtypes. However, one of the strengths of the study is that the findings obtained can serve as a reference for future studies focusing on more homogeneous subgroups within this heterogeneous structure. Another limitation is the retrospective single-center design of the study, which precludes causal inferences. On the other hand, it is thought that the results obtained will pave the way for further studies investigating the molecular mechanisms of the relationship between systemic inflammatory parameters and the clinical course and prognosis of the tumor. Finally, the lack of external validation, the absence of calibration analysis, the lack of longitudinal survival validation, and the modest predictive performance represent the notable limitations of the study. Due to the study’s retrospective design and its unsuitability for survival analyses, a calibration analysis could not be performed, and the agreement between the estimated probabilities and the observed results is uncertain; this issue should be addressed in future prospective studies. Despite these limitations, the comparative evaluation of multiple inflammation indices constitutes a significant strength.

## 5. Conclusions

The neutrophil count, NLR, PIV, SII, and SIRI may contribute moderately to the prediction of the clinical diagnosis and prognosis in brain tumor patients. Furthermore, a moderate association can be said to exist between inflammation and the clinical course and the prognosis of brain tumors. However, given the retrospective design of the study, the lack of external validation, and the moderate AUC values, further studies in more homogeneous subgroups in terms of variables such as tumor type, tumor location, and adjuvant therapy are needed to make these findings more specific and generalizable, and further molecular studies are needed to elucidate the underlying biological mechanisms of this relationship.

## Figures and Tables

**Table 1:** Characteristics and clinical data of the case group.

Parameter			Case Group *n* = 99 *
Tumor types *n* (%)		High-grade diffuse gliomas	26 (26.26)
		Low-grade diffuse gliomas	9 (9.09)
		Glioblastoma (IDH-wildtype)	6 (6.06)
		Pituitary adenoma	6 (6.06)
		Fibrous meningioma	5 (5.05)
		Adenocarcinoma metastasis	8 (8.08)
		Malignant epithelial tumor metastasis	13 (13.13)
		Neuroendocrine carcinoma metastasis	4 (4.04)
		Other	22 (22.22)
Tumor localization *n* (%)		Pituitary gland	6 (6.06)
		Right frontal	14 (14.14)
		Left frontal	14 (14.14)
		Right parietal	7 (7.07)
		Left parietal	12 (12.12)
		Right temporal	5 (5.05)
		Left temporal	10 (10.10)
		Left frontoparietal	7 (7.07)
		Left temporoparietal	6 (6.06)
		Other locations	18 (18.18)
Tumor size (mm) X ± SD		Anterior–posterior	37.72 ± 18.03
		Transverse	33.24 ± 16.92
		Craniocaudal	31.6 ± 17.71
Motor weakness *n* (%)		No	69 (69.70)
		Yes	30 (30.30)
Speech impairment *n* (%)		No	91 (91.92)
		Yes	8 (8.08)
Duration of surgery (min) X ± SD			96.72 ± 17.83
Intraoperative blood loss (mL) X ± SD			205.35 ± 70.21
Glasgow Coma Scale on postoperative day 1 X ± SD			14.62 ± 0.96
Ki-67 proliferation index X ± SD			16.16 ± 16.04
New neurological deficit *n* (%) (Postoperative day 1)		No	77 (77.78)
		Yes	22 (22.22)
3-month *n* (%)	Neurological deficit	No	68 (71.58)
		Yes	27 (28.42)
	Karnofsky Performance Status (KPS) X ± SD		82.84 ± 15.34
	Residual tumor	No	95 (100)
		Yes	0
	Recurrence	No	83 (87.37)
		Yes	12 (12.63)
	Adjuvant therapy	No	40 (42.11)
		Chemotherapy	48 (50.53)
		Radiotherapy	2 (2.11)
		Radiotherapy + chemotherapy	5 (5.26)
6-month *n* (%)	Neurological deficit	No	66 (69.47)
		Yes	29 (30.53)
	Karnofsky Performance Status (KPS) X ± SD		85.16 ± 16.69
	Residual tumor	No	93 (97.89)
		Yes	2 (2.11)
	Recurrence	No	82 (86.32)
		Yes	13 (13.68)
	Adjuvant therapy	No	39 (41.05)
		Chemotherapy	49 (51.58)
		Radiotherapy + chemotherapy	7 (7.37)
Re-surgery *n* (%)		No	78 (82.11)
		Yes	17 (17.89)

* Four patients died during the postoperative follow-up period. Therefore, the 3- and 6-month data were evaluated based on a total sample size of 95 patients. X ± SD: Mean ± Standard Deviation, IDH: Isocitrate Dehydrogenase.

**Table 2:** Laboratory findings of groups.

Parameter	Case Group *n* = 99	Control Group *n* = 99	*p*
Neutrophil (×10^9^/L)	7.84 ± 4.34	5.23 ± 1.96	<0.001
Lymphocyte (×10^9^/L)	1.95 ± 1.21	1.97 ± 0.81	0.296
Monocyte (×10^9^/L)	0.66 ± 0.43	0.64 ± 0.23	0.406
Platelet (×10^9^/L)	273.89 ± 88.51	257.35 ± 89.41	0.127
NLR	5.39 ± 4.12	3.33 ± 3.15	<0.001
PLR	185.15 ± 124.99	153.32 ± 96.58	0.092
MLR	0.39 ± 0.29	0.36 ± 0.18	0.885
PIV	1111.89 ± 1700.79	559 ± 590.26	0.021
SII	1551.77 ± 1488.45	887.39 ± 925.24	<0.001
SIRI	3.63 ± 4.53	2.05 ± 1.76	0.022

NLR: neutrophil/lymphocyte ratio, MLR: monocyte/lymphocyte ratio, PLR: Platelet/lymphocyte ratio, PIV: Pan-immune-inflammation value, SII: Systemic immune inflammation index, SIRI: Systemic inflammation response index.

**Table 3:** Results of logistic regression and ROC analysis of parameters used to assess the presence of brain tumors.

Parameters	Neutrophil	NLR	PIV	SII	SIRI
Odds ratio (95% CI)	1.329 (1.182–1.495)	1.195 (1.084–1.317)	1.049 (1.012–1.088) *	1.052 (1.021–1.083) *	1.200 (1.056–1.364)
*p* (Logistic regression)	<0.001	<0.001	0.009	0.001	0.005
AUC (95% CI)	0.696 (0.622–0.771)	0.655 (0.577–0.734)	0.595 (0.514–0.675)	0.652 (0.575–0.729)	0.594 (0.514–0.674)
Cut-off	7.43	4.05	452.28	726.00	3.27
*p* (ROC)	<0.001	<0.001	0.021	<0.001	0.022
Sensitivity % (95% CI)	50.51 (40.69–60.33)	51.52 (41.80–61.12)	62.63 (52.79–71.52)	65.66 (55.88–74.27)	34.34 (25.73–44.12)
Specificity % (95% CI)	88.89 (82.68–95.10)	83.84 (75.35–89.80)	60.61 (50.76–69.66)	63.64 (53.82–72.44)	87.88 (80.00–92.93)
PPV % (95% CI)	81.97 (71.61–92.33)	76.12 (64.67–84.73)	61.39 (51.64–70.30)	64.36 (54.65–73.01)	73.91 (59.74–84.40
NPV % (95% CI)	64.23 (54.81–73.65)	63.36 (54.84–71.12)	61.86 (51.91–70.90)	64.95 (55.05–73.71)	57.24 (49.29–64.83)

*: It is scaled in increments of 100 units. NLR: neutrophil/lymphocyte ratio, PIV: Pan-immune-inflammation value, SII: Systemic immune inflammation index, SIRI: Systemic inflammation response index, AUC: Area under curve, PPV: Positive predictive value, NPV: Negative predictive value.

**Table 4:** Results of correlation analysis of neutrophil, NLR, PIV, SII, and SIRI parameters of the case group with continuous and ordinal variables.

Parameter		Neutrophil		NLR		PIV		SII		SIRI	
		r	*p*	r	*p*	r	*p*	r	*p*	r	*p*
Tumor size	Anterior–posterior	0.179	0.077	0.183	0.070	0.141	0.164	0.114	0.260	0.222	0.028
	Transverse	0.105	0.303	0.099	0.327	0.099	0.330	0.068	0.503	0.143	0.157
	Craniocaudal	0.166	0.101	0.142	0.160	0.135	0.181	0.122	0.229	0.189	0.061
Duration of surgery		−0.134	0.187	−0.076	0.453	−0.182	0.071	−0.028	0.780	−0.218	0.030
Intraoperative blood loss		0.169	0.095	0.215	0.033	0.073	0.473	0.218	0.030	0.118	0.243
Glasgow Coma Scale		−0.175	0.083	−0.072	0.478	−0.085	0.405	−0.076	0.457	−0.081	0.427
Ki-67 proliferation index		0.122	0.296	0.148	0.204	0.053	0.653	0.140	0.232	0.068	0.563
KPS (3 months)		−0.287	0.005	−0.224	0.029	−0.264	0.010	−0.200	0.052	−0.290	0.004
KPS (6 months)		−0.236	0.021	−0.195	0.058	−0.216	0.035	−0.191	0.063	−0.214	0.037

NLR: neutrophil/lymphocyte ratio, PIV: Pan-immune-inflammation value, SII: Systemic immune inflammation index, SIRI: Systemic inflammation response index, KPS: Karnofsky performance status.

**Table 5:** Neutrophil, NLR, PIV, SII, and SIRI values of subgroups created according to clinical data of the case group.

Parameter	Neutrophil	NLR	PIV	SII	SIRI
**Tumor types**					
High-grade diffuse gliomas *n* = 26	8.45 ± 4.22	5.36 ± 3.95	859.41 ± 762.14	1436.08 ± 1128.21	2.96 ± 1.83
Low-grade diffuse gliomas *n* = 9	8.05 ± 3.67	6.83 ± 5.69	904.75 ± 912	2014.54 ± 1967.02	3.41 ± 3.13
Glioblastoma (IDH-wildtype) *n* = 6	3.99 ± 0.64	4.38 ± 3.09	212.26 ± 59.96	672.06 ± 236.01	1.22 ± 0.38
Pituitary adenoma *n* = 6	3.75 ± 0.64	1.61 ± 0.45	174.9 ± 65.58	357.98 ± 72.51	0.8 ± 0.37
Fibrous meningioma *n* = 5	4.13 ± 1.24	2.28 ± 0.83	361.6 ± 248.87	525.52 ± 301.69	1.53 ± 0.79
Adenocarcinoma metastasis *n* = 8	10.27 ± 2.33	8.7 ± 3	2001.58 ± 1879.26	2798.18 ± 1371.72	5.89 ± 4.07
Malignant epithelial tumor metastasis *n* = 13	7.57 ± 3.33	4.38 ± 2.89	1132.26 ± 1800.47	1311.36 ± 1096.13	3.76 ± 5.11
Neuroendocrine carcinoma metastasis *n* = 4	7.3 ± 1.75	7.92 ± 4.55	650.45 ± 217.71	2430.66 ± 1990.81	2.61 ± 0.99
Other *n* = 22	9.44 ± 5.88	5.78 ± 4.6	1914.74 ± 2776.66	1826.95 ± 1896.42	5.7 ± 7.43
***p***	<0.001	0.009	0.001	0.002	0.008
**Tumor localization**					
Pituitary gland *n* = 6	3.75 ± 0.64	1.61 ± 0.45	174.9 ± 65.58	357.98 ± 72.51	0.8 ± 0.37
Right frontal *n* = 14	8.04 ± 3.07	5.8 ± 4.4	1176.56 ± 1640.37	1901.57 ± 1770.42	3.43 ± 3.72
Left frontal *n* = 14	9.04 ± 4.76	5.43 ± 2.98	1052.58 ± 1130.53	1854.69 ± 1355.17	3.54 ± 4.24
Right parietal *n* = 7	5.83 ± 2.48	4.06 ± 4.19	416.2 ± 288.1	1071.36 ± 1177.2	1.57 ± 0.94
Left parietal *n* = 12	6.98 ± 4.77	5.46 ± 5.81	2060.9 ± 2848.21	1941.16 ± 2406.59	5.58 ± 6.87
Right temporal *n* = 5	7.28 ± 1.1	4.07 ± 1.37	851.3 ± 364.94	964.17 ± 341.66	3.58 ± 1.38
Left temporal *n* = 10	10.01 ± 5.07	7.63 ± 5.1	718.52 ± 576.1	1737.85 ± 1598.5	3.12 ± 2.21
Left frontoparietal *n* = 7	4.87 ± 2.37	4.95 ± 2.96	335.59 ± 345.9	1153.76 ± 892.95	2.06 ± 2.3
Left temporoparietal *n* = 6	11.03 ± 1.91	8.05 ± 2.77	1531.41 ± 1247.73	1880.1 ± 312.99	5.18 ± 2.13
Other locations *n* = 18	8.54 ± 5.48	5.18 ± 4.05	1510.87 ± 2431.59	1474.44 ± 1388.82	4.7 ± 6.82
***p***	0.003	0.039	0.018	0.029	0.026
**Motor weakness**					
No *n* = 69	7.38 ± 4.54	4.63 ± 3.76	911.39 ± 1353.85	1283.06 ± 1293.44	3.12 ± 4.05
Yes *n* = 30	8.9 ± 3.68	7.13 ± 4.44	1573.01 ± 2271.97	2169.8 ± 1731.19	4.8 ± 5.38
***p***	0.019	0.003	0.019	0.001	0.070
**Speech impairment**					
No *n* = 91	7.64 ± 4.2	5.18 ± 3.87	978.24 ± 1359.96	1445.23 ± 1288.31	3.36 ± 4.04
Yes *n* = 8	10.11 ± 5.51	7.75 ± 6.12	2632.08 ± 3720.91	2763.63 ± 2802.01	6.69 ± 8.16
***p***	0.147	0.317	0.120	0.114	0.281
**New neurological deficit (Postoperative day 1)**					
No *n* = 77	7.52 ± 4.5	4.97 ± 3.95	1020.44 ± 1466.32	1356.13 ± 1315.54	3.5 ± 4.33
Yes *n* = 22	9 ± 3.56	6.85 ± 4.43	1431.96 ± 2362.2	2236.53 ± 1855.83	4.09 ± 5.27
***p***	0.036	0.030	0.069	0.004	0.429
**Neurological deficit (3 months)**					
No *n* = 68	7.34 ± 4.56	4.74 ± 4.08	1039.43 ± 1671.14	1377.42 ± 1552.29	3.37 ± 4.53
Yes *n* = 27	8.89 ± 2.44	6.75 ± 3.59	1100.7 ± 1261.32	1858.43 ± 1059.51	3.89 ± 3.62
***p***	0.005	0.003	0.039	0.002	0.089
**Recurrence (3 months)**					
No *n* = 83	7.73 ± 4.19	5.26 ± 4.07	1086.21 ± 1619.34	1549.75 ± 1513.39	3.64 ± 4.5
Yes *n* = 12	8.11 ± 3.72	5.62 ± 3.91	853.73 ± 1085.27	1267.8 ± 766.42	2.69 ± 2.25
***p***	0.516	0.667	0.467	0.703	0.929
**Adjuvant therapy (3 months)**					
No *n* = 40	6.41 ± 3.33	4.3 ± 3.99	599.17 ± 661.12	1176.62 ± 1316.29	2.22 ± 1.95
Chemotherapy *n* = 48	8.4 ± 4.16	5.76 ± 3.83	1389.93 ± 1994.84	1629.43 ± 1466.32	4.56 ± 5.47
Radiotherapy *n* = 2	13.25 ± 0.64	5.1 ± 0.09	3113.84 ± 233.14	2231.75 ± 155.81	7.12 ± 0.16
Radiotherapy + chemotherapy *n* = 5	10.64 ± 6.22	9.09 ± 4.88	697.74 ± 633.6	2820.22 ± 1710.06	2.49 ± 2.04
***p***	0.012	0.017	0.028	0.005	0.032
**Neurological deficit (6 months)**					
No *n* = 66	7.27 ± 4.61	4.8 ± 4.13	1051.65 ± 1695.14	1399.49 ± 1570.65	3.39 ± 4.6
Yes *n* = 29	8.93 ± 2.36	6.48 ± 3.61	1068.65 ± 1221.33	1775.03 ± 1067.55	3.81 ± 3.5
***p***	0.002	0.010	0.037	0.006	0.067
**Recurrence (6 months)**					
No *n* = 82	7.49 ± 4.28	5.23 ± 4.18	1063.39 ± 1640.09	1449.87 ± 1448.94	3.59 ± 4.55
Yes *n* = 13	9.61 ± 2.21	5.81 ± 2.99	1015.51 ± 941.73	1919.49 ± 1371.53	3.04 ± 1.88
***p***	0.007	0.274	0.166	0.061	0.309
**Adjuvant therapy (6 months)**					
No *n* = 39	6.97 ± 3.46	4.28 ± 3.6	776.59 ± 853.28	1254.94 ± 1309.89	2.62 ± 2.17
Chemotherapy *n* = 49	8.38 ± 4.15	5.92 ± 4	1357.52 ± 1984.95	1642.09 ± 1462.22	4.47 ± 5.45
Radiotherapy + chemotherapy *n* = 7	8.07 ± 6.72	6.72 ± 5.68	513.51 ± 605.5	2062.48 ± 1903.74	1.85 ± 1.99
***p***	0.216	0.099	0.176	0.132	0.175
**Re-surgery**					
No *n* = 78	7.65 ± 4.35	5.32 ± 4.11	1086.06 ± 1673.4	1480.36 ± 1465.71	3.66 ± 4.63
Yes *n* = 17	8.36 ± 2.84	5.27 ± 3.76	922.76 ± 885.81	1669.09 ± 1350.62	2.86 ± 2.03
***p***	0.176	0.823	0.356	0.303	0.662

## Data Availability

The datasets generated and analyzed during the current study are not publicly available due to patient privacy regulations but are available from the corresponding author upon reasonable request and with appropriate ethical approvals.

## Abbreviations

The following abbreviations are used in this manuscript:

NLR	Neutrophil/lymphocyte ratio
PIV	Pan-immune-inflammation value
SII	Systemic immune inflammation index
SIRI	Systemic inflammation response index
KPS	Karnofsky performance status
MLR	Monocyte/lymphocyte ratio
PLR	Platelet/lymphocyte ratio
AUC	Area under curve
ROC	Receiver operating characteristic
WHO	World Health Organization
IDH	Isocitrate dehydrogenase
